# Intelligent CpG nanoplatforms for targeted cancer immunotherapy and immune remodeling

**DOI:** 10.3389/fphar.2026.1826929

**Published:** 2026-06-12

**Authors:** Huixia Wang, Yangming Zhang, Mengjiao Zhou

**Affiliations:** 1 The People’s Hospital of Danyang, Affiliated Danyang Hospital of Nantong University, Danyang, Jiangsu Province, China; 2 School of Pharmacy, Nantong University, Nantong, Jiangsu Province, China

**Keywords:** cancer immunotherapy, CpG oligonucleotides, immunogenic cell death, intelligent nanoplatforms, tumor immune microenvironment

## Abstract

CpG oligonucleotides are potent immunoadjuvants that hold great promise for cancer immunotherapy, but their clinical use is limited by *in vivo* instability, off-target toxicity, and poor tumor targeting. Intelligent nanoplatforms offer effective solutions to these challenges by protecting CpG, improving delivery precision, and enabling on-demand cargo release. This review summarizes four major design strategies: biomimetic/biohybrid systems for targeted delivery, stimuli-responsive nanocarriers for controlled release, structural engineering for enhanced immune activation, and combinatorial therapy for systemic immune remodeling. These nanoplatforms not only improve CpG stability and tumor-targeting capability but also reshape the tumor immune microenvironment, convert “cold” tumors to “hot” phenotypes, and induce long-term immune memory. We highlight the design principles, action mechanisms, key advantages, and inherent limitations of current intelligent CpG-based nanosystems, aiming to advance the development and clinical translation of CpG nanotherapeutics for more effective and personalized cancer immunotherapy.

## 1 Introduction

The rise of cancer immunotherapy has revolutionized the fight against malignancies (Guo et al., 2023; Chen et al., 2024; Yu et al., 2024; Yu and Yao, 2024; Cai et al., 2025; Wang Y. et al., 2025). Within this paradigm, immunoadjuvants play a pivotal role, acting as both “alarms” and “amplifiers” to initiate and potentiate antigen-specific immune responses (Banstola et al., 2020; Cao et al., 2025; Li et al., 2020a; Wang Z. et al., 2025; Xu et al., 2022; Yan et al., 2025; Yang et al., 2025; Yu et al., 2025; Zhong et al., 2025; Zhou et al., 2021). Among them, CpG oligodeoxynucleotides (CpG ODNs) have garnered intense interest due to their unique mechanism and robust immunostimulatory capacity (Yu et al., 2022; Zhang et al., 2023a; Zhang et al., 2023b; Gong et al., 2024; Murata et al., 2024; Qureshi et al., 2024). CpG function is tightly regulated by several interrelated factors critical to immunological rigor and translational relevance, including biodistribution, intracellular trafficking, cell-type specificity, and dose-dependent immunotoxicity (Kaneko et al., 2025; Ji et al., 2024). First, unformulated CpG ODNs can be rapidly degraded by nucleases, resulting in an extremely short half-life (Ji et al., 2023; Zhao et al., 2025a; Liu S. et al., 2023; Takano et al., 2023; Zhong et al., 2023; Zhang H. et al., 2024). Second, CpG ODNs must reach endosomal Toll-like receptor 9 (TLR9) to exert activity, and inefficient endocytosis or failed endosomal escape significantly impairs bioavailability (Li M. et al., 2024). Third, CpG recognizes TLR9 on human B cells and plasmacytoid dendritic cells (pDCs), while TLR9 activation in T cells and monocytes/macrophages further drives cytokine and chemokine secretion (Xie et al., 2024; Xiong et al., 2024; Zhang Z. et al., 2024; Chen et al., 2025; Milligan et al., 2025). These cellular events promote the production of pro-inflammatory mediators, establish Th1-biased immunity, enhance pDC antigen presentation, and stimulate robust CD8^+^ T cell priming and cytotoxic T lymphocytes (CTLs) cytotoxicity. However, off-target immune cell activation may also trigger aberrant inflammatory responses (Shi et al., 2025; Xiong et al., 2025; Han et al., 2026; Yu et al., 2026; Yuan et al., 2026). Fourth, low doses of CpG fail to elicit effective anti-tumor immunity, whereas high doses or uncontrolled CpG release carry severe risks of systemic inflammation or cytokine storms (Zhang et al., 2017; Scully et al., 2022; Lu et al., 2023; Bell et al., 2025; Hu et al., 2025; Wei et al., 2025; Li et al., 2026; Yang et al., 2023). Given the heterogeneous responses of distinct immune cell subsets to CpG, targeted and controlled delivery is urgently needed to minimize off-target effects and avoid systemic immune disorders.

In recent years, nanomedicine and nanotechnology provide a unique basis for targeted delivery. Nanocarriers can protect nucleic acids from nuclease degradation, prolong blood circulation, enhance tumor accumulation *via* the enhanced permeability and retention (EPR) effect, and realize spatiotemporally controlled release (Goldberg, 2019; Lou et al., 2019; Asadujjaman et al., 2020; Yang et al., 2020; Zeng et al., 2023; Omole et al., 2024; Pan et al., 2024; Zhou Y. et al., 2024). At the molecular level, nanoplatforms regulate intracellular trafficking of CpG ODNs, promote endosomal escape to activate TLR9 signaling, and optimize the interaction between adjuvants and immune cells (He et al., 2023; Hu et al., 2023; Ishaqat et al., 2023; Li L. et al., 2023; Li X. et al., 2023). This approach significantly enhances the magnitude and specificity of immune responses and offers a powerful strategy to overcome multiple limitations associated with conventional immunotherapies (Xin et al., 2025; Yang et al., 2025a; Yang et al., 2025b). For precision targeting, biomimetic camouflage strategies stand out. Coating nanoparticles with tumor cell membranes endows them with homotypic targeting ability while presenting a full repertoire of tumor-associated antigens, effectively merging vaccine and delivery functions (Sun et al., 2017; Wu et al., 2019; Sun et al., 2020; Meng et al., 2021; Pei et al., 2021; Ren et al., 2021; Arduino et al., 2024; Zhou M. et al., 2024; Zou et al., 2024). Hybrid membranes derived from fused macrophage-tumor cells combine targeting with built-in co-stimulatory signals, greatly enhancing antigen presentation. Red blood cell membrane cloaking, meanwhile, prolongs circulation and leverages the EPR effect for passive tumor accumulation (Tang et al., 2016; Liu et al., 2018; Xuan et al., 2018; Chen et al., 2020; Yuan et al., 2025). Ligand-receptor approaches remain equally relevant: mannose-functionalized carriers target mannose receptors on dendritic cells (DCs) (Luo et al., 2024), while hyaluronic acid directs delivery to CD44-overexpressing tumor and immune cells (Zhao et al., 2025b; Li et al., 2020b; Mo et al., 2021; Catania et al., 2023; Ghalehkhondabi et al., 2023; Kim et al., 2024). Recently, engineered bacterial outer membrane vesicles, natural nano-immunostimulants, have emerged as hybrid platforms that couple targeting with immune activation (Lin et al., 2025). Once at the target site, stimulus-responsive release mechanisms act as precise “on-switches” for CpG activity. These designs exploit distinctive features of the tumor microenvironment or external triggers (Ling et al., 2024). The synergy between active targeting and smart release forms the foundation of modern CpG nanoplatforms, enabling “precision delivery with on-demand activation.”

As design sophistication grows, CpG nanocarriers have transcended mere adjuvant delivery to become multifunctional “hubs” for combinatorial immunotherapy (Wang et al., 2016; Chen et al., 2018; Dong et al., 2019; Ming et al., 2020; He et al., 2021; Wang et al., 2021). Their true power lies in synergistic integration, *via* co-loading, sequential release, or combination regimens, with diverse therapeutic modalities (Zhang et al., 2021; We et al., 2022; Karthikeyan et al., 2023; Zhang X. et al., 2023; Jiang et al., 2025). Combining CpG with chemotherapy enables “immunochemotherapy”: chemotherapeutics inducing immunogenic cell death (ICD), releasing tumor antigens that, when paired with CpG’s adjuvant effect, potently prime antigen-specific T cells (Zhao S. et al., 2025). Similarly, coupling with physical modalities, such as photodynamic therapy or radiotherapy, yields abscopal effects: localized tumor killing and ICD synergize with CpG-driven systemic immunity to control both primary and distant metastatic lesions (Wang S. et al., 2025). Even more advanced strategies involve metabolic reprogramming or gene silencing to actively reverse immunosuppression, thereby creating a permissive niche for CpG-activated immune cells. Collectively, these approaches mark a strategic shift, from CpG as a supporting player to a central orchestrator of antitumor immunity.

Through such intelligent engineering, the therapeutic ambition of CpG nanoplatforms has expanded beyond tumor shrinkage to systemic immune remodeling. Successful treatment not only suppresses primary tumor growth but also reprograms the tumor immune microenvironment: boosting infiltration of cytotoxic CD8^+^ T cells and pro-inflammatory M1-like tumor-associated macrophages, while reducing immunosuppressive regulatory T cells (Tregs) and M2-like macrophages, effectively converting “cold” tumors into “hot” ones (Shi et al., 2023). Critically, these platforms can elicit durable systemic immunity and long-term immunological memory, preventing recurrence and metastasis. *In situ* vaccines based on aluminum salts or metal-phenolic networks harness tumor lysates as personalized antigens to sustain immune surveillance. Likewise, structurally optimized DNA origami vaccines co-delivered with antigens generate persistent T cell memory (Zeng Y. C. et al., 2024; Miyamoto et al., 2024). Looking ahead, CpG nanoplatforms hold immense promise in personalized medicine, especially when integrated with neoantigens, total tumor RNA, or patient-derived tumor cell membranes, to enable truly tailored, high-efficacy, low-toxicity immunotherapy.

**FIGURE 1:**
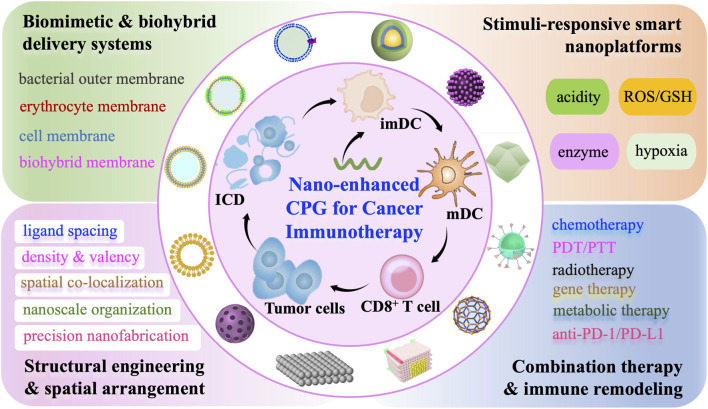
Schematic overview of the design strategy and antitumor mechanism of CpG-based nanoplatforms for cancer immunotherapy. This review is structured around four core design principles of CpG-based nanoplatforms: biomimetic and biohybrid delivery systems, stimuli-responsive smart nanoplatforms triggered by tumor microenvironmental cues, structural engineering and spatial arrangement design, and combination therapy and systemic immune remodeling with various therapeutic modalities. Abbreviations: ROS, reactive oxygen species; GSH, glutathione; PDT, photodynamic therapy; PTT, photothermal therapy; imDC, immature dendritic cell; mDC, mature dendritic cell.

In summary, the effective deployment of CpG ODNs has become a multidisciplinary endeavor spanning molecular immunology, materials science, nanotechnology, and clinical oncology. Cutting-edge research now focuses on developing “smart” nanovaccines capable of biological sensing, programmable decision-making, and multifunctional execution. To systematically map this rapidly evolving landscape, this review synthesizes recent advances in the design, delivery, and antitumor mechanisms of CpG nanovaccines, organized into four sections (Guo et al., 2023): Biomimetic and biohybrid delivery systems: how natural recognition modules, such as cell membranes and bacterial vesicles, enable precise targeting and immune modulation (Figure 1); (Chen et al., 2024) Stimuli-responsive smart nanoplatforms: how endogenous or exogenous triggers govern spatiotemporal CpG release and enable combination therapy (Yu et al., 2024); Structural engineering and spatial arrangement: how supramolecular chemistry and DNA nanotechnology optimize immune signal organization at the nanoscale to maximize receptor activation (Yu and Yao, 2024); Combinatorial therapy and systemic immune remodeling: how CpG platforms serve as integrative hubs to orchestrate multimodal treatments, ultimately driving robust, sustained antitumor immunity. By integrating these strategies, we aim to provide a comprehensive framework and forward-looking insights for the rational design of next-generation nanovaccines, more efficient, intelligent, and clinically translatable, for the future of cancer immunotherapy.

## 2 From smart delivery to integrated immunomodulation

Before exploring the design and application of CpG-based nanoplatforms, it is essential to clarify their clinical translation status. Table 1 summarizes representative CpG-derived immunotherapeutic agents in ongoing or completed clinical trials, outlining their formulation characteristics, applicable tumor types, and clinical development stages. Nevertheless, CpG immunoadjuvants face several critical bottlenecks, including poor *in vivo* stability, low targeting efficiency, and systemic toxicity. The integration of modern nanotechnology has fundamentally reframed this challenge as a multivariate “biological interface engineering” problem, with successful strategies consistently revolving around two core dimensions: spatial precision and temporal programmability (Nair et al., 2024). Spatial precision is achieved through biomimetic membrane cloaking, ligand functionalization, and other active targeting approaches (Varghese et al., 2019). These enable nanocarriers to act like “Trojan horses,” bypassing physiological barriers and selectively recognizing target cells. Temporal programmability, on the other hand, leverages responsiveness to intrinsic tumor microenvironment cues or external energy fields (*e.g.*, light, magnetic fields) to ensure therapeutic payloads are released precisely at the optimal moment for immune activation.

**TABLE 1:** Representative CpG-based immunotherapies in clinical trials for cancer immunotherapy.

Platform	Formulation	Cancer type	Clinical phase
Vidutolimod (CMP-001)	CpG-A encapsulated in Qβ phage virus-like particle	melanoma	Phase I (NCT02680184)
Tilsotolimod (IMO-2125)	synthetic dimeric CpG ODN	melanoma	Phase III (NCT03445533)
Nelitolimod (SD-101)	synthetic CpG-C ODN	melanoma, lymphoma	Phase II (NCT02521870)
MGN1703	dumbbell-shaped double stem-loop CpG	advanced solid tumors	Phase II (NCT02668770)
PF-3512676	CpG-B ODN with phosphorothioate backbone	melanoma, lymphoma	Phase III (terminated)

However, even the most advanced targeting and stimuli-responsive release systems may fall short against the complexity and heterogeneity of the immunosuppressive tumor microenvironment. The next level of innovation lies in elevating CpG nanoplatforms from “smart carriers” to systemic hubs for combination immunotherapy. This represents a second paradigm shift: moving beyond single-function delivery vehicles toward integrated platforms that combine multiple therapeutic modalities to actively reprogram the dynamic equilibrium between tumors and the immune system, essentially functioning as “integrated immunomodulatory regulators.” To systematically organize and clearly present the research advances covered in this review, Table 2 summarizes representative CpG-based nanoplatforms reported in recent years. It details their design strategies, physicochemical properties, performance advantages, and key applications in cancer immunotherapy.

**TABLE 2:** Representative recent research efforts on various CpG-based nanoplatforms for cancer immunotherapy.

Nanovaccine	Therapeutic method	Design strategies	Property and therapeutic effect	Immune outcome	Ref
FCM@4RM	CpG	Fused cytomembrane camouflaged	size = 219.4 ± 3.7 nm, zeta = −21.7 ± 0.8 mV, LE of CpG = 68.6%, LE of MET = 25.2%, 4T1 cells, 3T3 cells	DC maturation↑, CTL activation↑, M2→M1↑, PD-L1↓	Ji et al. (2023)
CpG@MSN-PEG/PEI@OMVs	CpG	Bacterial outer membrane vesicle cloaked	size = 54.0 ± 0.1 nm, zeta = −15.8 ± 1.08 mV, 4T1 and MC38 cells	DC maturation↑, M2→M1↑, IFN-γ↑, TGF-β↓	Lin et al. (2025)
PLGA-CpG@ID8-M	CpG	Tumor cell membranes cloaked	size ≈ 144 nm, zeta = −28.3 mV, ovarian cancer	M2→M1↑, CD47↓, antitumor immunity↑	Xiong et al. (2025)
ZLGCR	CpG	Red blood cell membrane cloaked	size ≈ 110.3 ± 8.4 nm, 4T1 cells	TME remodeling↑, DC maturation↑, CD8^+^ T↑	Ji et al. (2024)
Alum-CpG@Fe-Shikonin	ferroptosis + necroptosis + CpG	pH-responsive	LE of CpG = 87.5%, size ≈ 219.7 ± 63.7 nm, zeta = 7.0 ± 3.1 mV, 4T1 cells	ICD↑, DC maturation↑, CD8^+^ T↑	Shi et al. (2023)
BPNS@Mn^2+^/CpG	PTT + CDT + CpG	pH-responsive	adsorption of CpG = 53.9%, size ≈ 310 nm, zeta = -44.9 ± 2 mV, 4T1 cells	TLR9/STING↑, DC maturation↑, M2→M1↑, CD8^+^ T↑, ICD↑, TME remodeling↑	Ling et al. (2024)
FNC@NF	CpG	ROS-responsive	size ≈ 39.97 nm, zeta = −24.59 mV, hexagonal shape, pancreatic adenocarcinoma	TLR9↑, DC maturation↑, CD8^+^ T↑, immune evasion↓	Zhao et al. (2023)
DAPC-Fuc/CpG	ferroptosis + CpG	ROS-responsive	LE of CpG = 2.9 ± 0.1%, EE of CpG = 79.3 ± 0.4 %, size ≈ 70-80 nm, 4T1 cells	DC maturation↑, CD8^+^ T↑, IFN-γ↑	Gao et al. (2024)
DoriVac	CpG + anti-PD-L1	DNA origami	CpG is spaced (3.5 nm), dimensions = 35.0 × 22.5 × 27.0nm^3^, zeta = −3.3 ± 0.8 mV, B16F10 cells	DC maturation↑, Th1 polarization↑, CD8^+^ T↑, checkpoint synergism↑	Zeng Y. C. (2024)
MMOC/MMCC	CpG + anti-PD-L1	Tumor cell membrane proteins-encapsulated	Size of MMOC ≈ 142.5 ± 1.1 nm, EE of CpG = 100%, 4T1 cells	DC maturation↑, CD8^+^ T↑, CTL activation↑, PD-L1↓	Luo et al. (2024)
EaCpG	CpG	Extracellular matrix anchored	petal-like structures, B16F10 cells	DC maturation↑, CD8^+^ T↑, abscopal effect↑	Yang et al. (2023)
Mn@CpG@NLP	CpG	STING signaling pathway	size ≈ 150 nm, zeta = 6 mV, EE% of CpG = 99.8%, colon cancer stem cells	TLR9/STING↑, DC maturation↑, CD8^+^ T↑, cancer stem cells↓	Zhao et al. (2025a)
αPD-L1/CpG@MCL	CpG + anti-PD-L1 + hyperthermia	Thermo-immunotherapy	size ≈ 134 nm, zeta = −16.3 mV, B16F10 cells	M2→M1↑, CD8^+^ T↑, PD-L1 blockade↑, thermo-immunity↑	Kaneko et al. (2025)
HCNPs/DNPs	CpG + doxorubicin	pH-responsive release, CD44 targeting	size of DNPs ≈ 118.2 ± 15.3 nm, size of HCNPs ≈ 150.8 ± 8.6 nm, MC-38 cells	M2↓, memory T↑, ICD↑	Zhao et al. (2025b)
tNano-S&C	CpG + STAT3 siRNA	Chemoimmunotherapy	size ≈ 45 nm, neutral zeta potential, spherical vesicles, malignant glioma	STAT3↓, M2↓, Treg↓, ICD↑	Zhao S. et al. (2025)
PTC NVs@MNs	TdRNA + CpG	Dissolving microneedle patch	size ≈ 149.06 nm, zeta = 12.61 mV, transdermal, triple-negative breast cancer	DC maturation↑, CD8^+^ T↑, immune memory↑	Wang J. et al. (2025)
Ce6/CpG@Lip-TD	Ce6 + CpG	Biopeptide-modified liposomes	size ≈ 125 nm, zeta = 26.1 ± 2.04 mV, B16F10 cells, superficial tumors	ICD↑, DC maturation↑, CD8^+^ T↑, abscopal effect↑	Wang S. et al. (2025)

Abbreviations: CDT, chemodynamic therapy; Ce6, chlorin e6; EE, encapsulation efficiency; IFN-γ, interferon-γ; LE, loading efficiencies; MET, metformin; PTT, photothermal therapy; ROS, reactive oxygen species; STAT3, signal transducer and activator of transcription 3; STING, stimulator of interferon genes; TdRNA, tumor-derived total RNA; TME, tumor microenvironment; Treg, regulatory T cell.

## 3 Biomimetic and biohybrid nanodelivery systems

**FIGURE 2:**
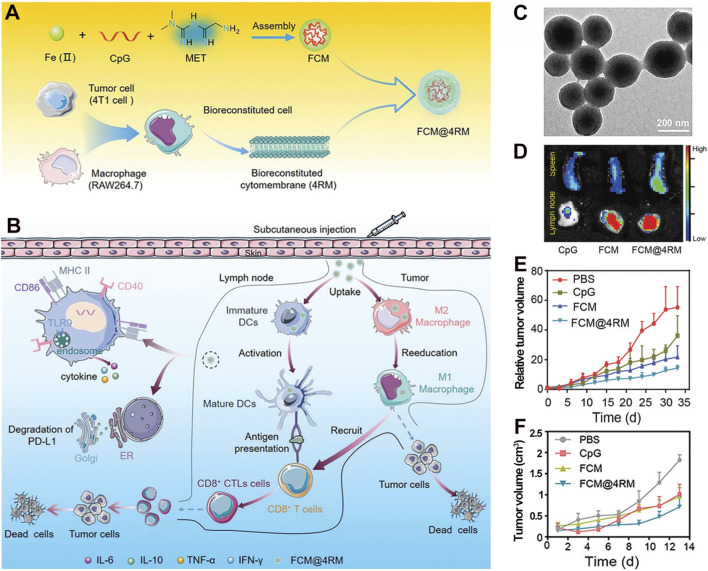
**(A)** Schematic illustration of the design strategy for the FCM@4RM nanoplatform. **(B)** Immune-mediated antitumor mechanisms of the FCM@4RM nanoplatform. **(C)** TEM image of FCM nanoparticles. **(D)** Comparison of accumulation in the spleen and lymph nodes among CpG, FCM, and FCM@4RM groups. **(E)** Relative tumor volume changes in mice across different treatment groups. **(F)** Tumor growth curves in mice following various treatments. Reproduced with permission from Ji et al. (2023). Copyright (2023), Wiley-VCH GmbH.

Biomimetic and biohybrid strategies aim to overcome key limitations of synthetic nanomaterials, such as poor biocompatibility, rapid immune clearance, and limited functionality (Cao et al., 2022; Dhas et al., 2022; Du et al., 2022; Pat et al., 2022; Wang et al., 2022; Zhang et al., 2022; Zhao et al., 2022). They achieve this by borrowing or integrating natural biological components (Huang et al., 2023; Jiang et al., 2024; Wang et al., 2024; Yang C. et al., 2024; Liu et al., 2025). By cloaking nanoparticles with “biological identity tags” like cell membranes or bacterial vesicles, CpG-based platforms can achieve precise targeting, prolonged circulation, and enhanced immunomodulatory capabilities. This paves the way for more effective and safer immunotherapies. A compelling example is the “core–shell” nanovaccine FCM@4RM developed by Ji et al. (Ji et al., 2023). The core (FCM) is formed through the self-assembly of Fe(II), CpG, and metformin (MET), combining two functions in one: TLR9 activation (*via* CpG) and PD-L1 blockade (*via* MET). The shell consists of a hybrid membrane derived from fused 4T1 tumor cells and RAW264.7 macrophages (termed 4RM) (Figure 2A). This design significantly promoted dendritic cell maturation and antigen presentation. In a 4T1 tumor model, FCM@4RM triggered robust antitumor immunity by simultaneously activating CTLs and inhibiting the PD-L1 checkpoint (Figure 2B). Transmission electron microscopy (TEM) confirmed the spherical morphology of the FCM nanoparticles (Figure 2C). Fluorescence imaging further showed efficient delivery of the vaccine to key immune organs, the spleen and draining lymph nodes, demonstrating excellent targeting and accumulation (Figure 2D). *In vivo* studies revealed strong suppression of tumor growth (Figure 2E). Notably, in a bilateral tumor model, FCM@4RM selectively targeted and treated only the 4T1 tumors, highlighting the homotypic targeting conferred by the tumor cell membrane (Figure 2F). This approach offers a promising avenue for personalized immunotherapy and precision drug delivery.

**FIGURE 3:**
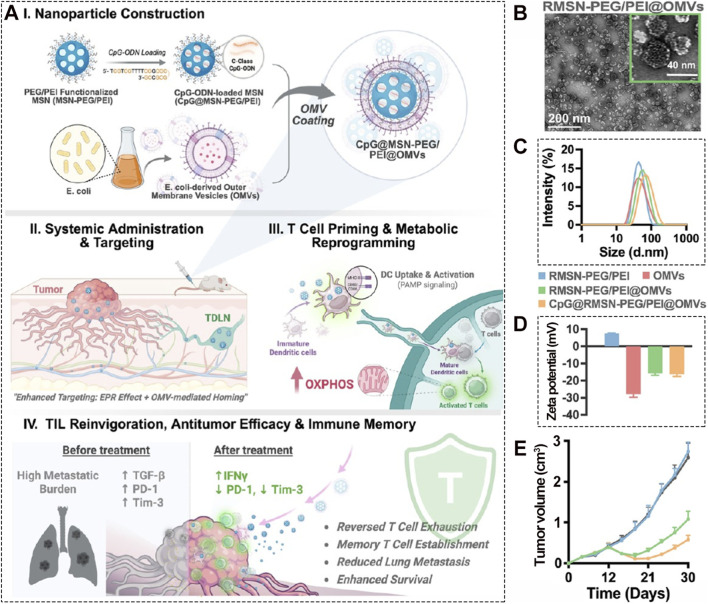
**(A)** Schematic illustration of the design and immune-mediated antitumor mechanism of the CpG@MSN-PEG/PEI@OMVs nanoplatform. **(B)** TEM image of RMSN-PEG/PEI@OMVs. **(C)** Hydrodynamic diameters of various nanomaterials determined by DLS. **(D)** Zeta potentials of different nanomaterials. **(E)** Antitumor efficacy of different treatment groups. Reproduced with permission from Lin et al. (2025).

Despite the advantages of cell membrane coating, the resulting immune activation can sometimes be insufficient. To address this, researchers have turned to bacterial components with inherent, potent adjuvant properties (Gerritzen et al., 2017; Farjadian et al., 2018; Sartorio et al., 2021; Xue et al., 2022; Liu N. et al., 2023; Singh et al., 2024; Fan et al., 2025). For example, Lin et al. engineered a hybrid platform, CpG@MSN-PEG/PEI@OMVs, by loading CpG into mesoporous silica nanoparticles (MSNs) and enveloping them with outer membrane vesicles (OMVs) from *E. coli* (Figure 3A) (Lin et al., 2025). This design merges the precise drug-loading capacity of synthetic nanoparticles with the natural immunogenicity of OMVs. After intravenous injection, the system efficiently accumulated in tumors. More importantly, the OMV shell and released CpG acted synergistically to strongly activate DCs and polarize macrophages toward the pro-inflammatory M1 phenotype. Beyond direct tumor targeting, this platform systemically reprogrammed the immunosuppressive tumor microenvironment, leading to remarkable therapeutic outcomes across multiple tumor models. TEM images verified the spherical core-shell nanostructure (Figure 3B), while dynamic light scattering (DLS) confirmed changes in hydrodynamic size consistent with successful coating (Figure 3C). Zeta potential measurements showed a charge reversal, from positive (MSN-PEG/PEI) to negative, after OMV encapsulation, further supporting successful hybridization (Figure 3D). In 4T1 tumor-bearing mice, the platform significantly inhibited tumor growth (Figure 3E), demonstrating that OMV coating enhances both CpG delivery and overall immunostimulation.

**FIGURE 4:**
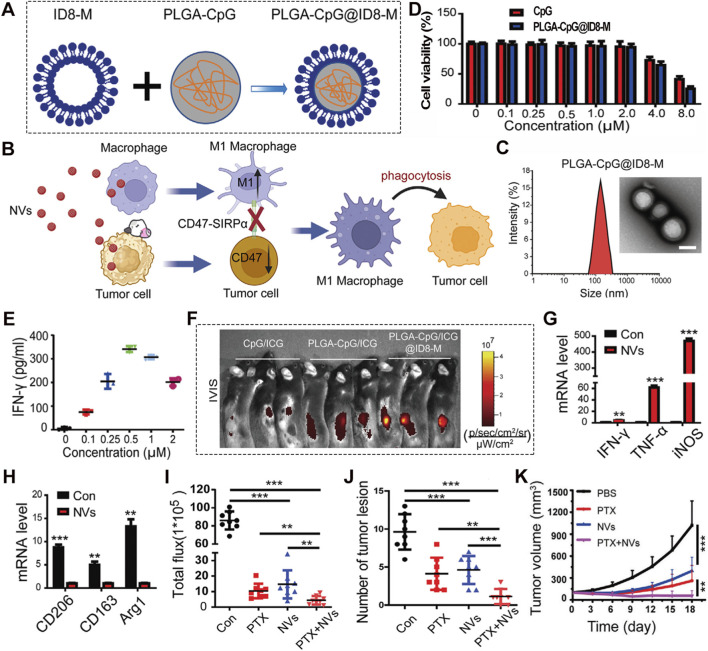
**(A)** Schematic illustration of the design strategy for the PLGA-CpG@ID-M nanoplatform. **(B)** Schematic illustration of the antitumor mechanism of nanovaccines. **(C)** TEM image and DLS size distribution of PLGA-CpG@ID-M nanoparticles. **(D)** Cell viability after treatment with CpG or PLGA-CpG@ID-M nanoparticles at various concentrations. **(E)** IFN-γ production induced by PLGA-CpG@ID-M nanoparticles at different concentrations. **(F)** Comparison of tumor accumulation among different control groups. **(G,H)** mRNA expression levels of canonical M1 and M2 macrophage markers in control versus nanovaccine-treated groups. **(I)** Antitumor efficacy across different treatment groups. **(J)** Quantification of tumor lesions in each experimental group. **(K)** Tumor volume changes over time following administration of various treatments. Reproduced with permission from Xiong et al. (2025).

While bacterial OMVs provide broad innate immune activation, another critical strategy involves precisely reprogramming specific immunosuppressive cell populations within the tumor microenvironment. Tumor-associated macrophages (TAMs), especially the M2-polarized subset, are prime targets. For ovarian cancer, which features abundant M2 TAMs, Xiong et al. developed PLGA-CpG@ID8-M, a nanovaccine composed of CpG-loaded PLGA nanoparticles coated with membranes from ID8 ovarian tumor cells (ID8-M) (Figure 4A) (Xiong et al., 2025). The ID8-M coating not only enabled homologous tumor targeting but also supplied tumor-associated antigens. Upon uptake by TAMs, the released CpG effectively repolarized them toward the antitumor M1 phenotype (Figure 4B). DLS and TEM analyses showed particle sizes of approximately 144 nm and 115 nm, respectively (Figure 4C). Cytotoxicity assays confirmed good biocompatibility at concentrations up to 2 µM (Figure 4D). ELISA results indicated peak IFN-γ secretion at 0.5 µM CpG (Figure 4E). *In vivo* imaging demonstrated significantly enhanced tumor accumulation due to the cell membrane cloak (Figure 4F). Further analysis revealed upregulation of M1 markers (Figure 4G) and downregulation of M2 markers (Figure 4H), thereby reducing tumor immune evasion. When combined with the chemotherapeutic paclitaxel (PTX), the nanovaccine achieved superior tumor suppression (Figure 4I) and markedly reduced metastatic burden (Figure 4J). Survival studies in an ovarian cancer model confirmed the strongest antitumor effect in the combination group (Figure 4K), offering a novel therapeutic strategy for this challenging malignancy.

**FIGURE 5:**
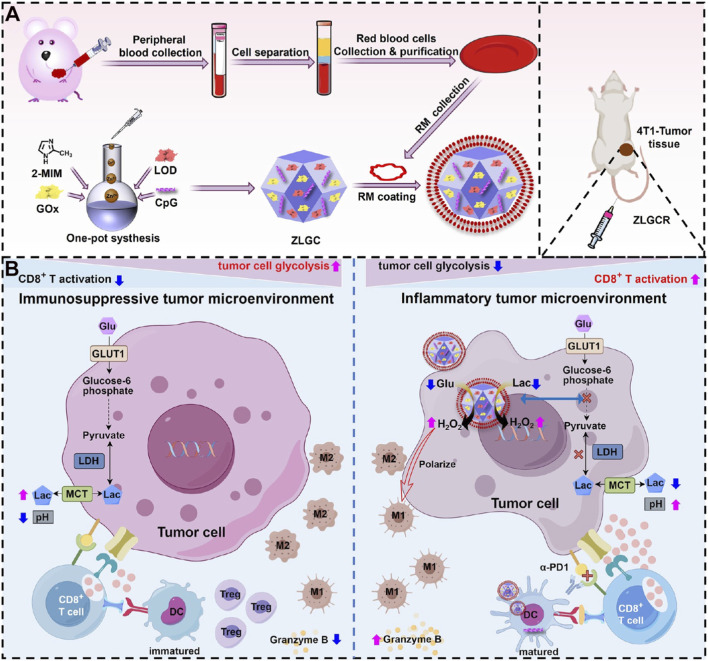
**(A)** Schematic illustration of the ZLGCR nanoplatform design. **(B)** Schematic illustration of the combination antitumor strategy using ZLGCR and anti-PD-1 antibody. Reproduced with permission from Ji et al. (2024). Copyright (2024), American Chemical Society.

Reprogramming TAMs addresses cellular-level immunosuppression, but the tumor microenvironment is also shaped by abnormal metabolic conditions that profoundly suppress immune responses. Therefore, simultaneously modulating metabolism and immunity represents a more holistic therapeutic approach. Chen et al. developed an integrated platform called ZLGCR (Figure 5A), which co-encapsulates glucose oxidase, lactate oxidase, and CpG within a zeolitic imidazolate framework-8 (ZIF-8) core, all camouflaged with red blood cell membranes (Ji et al., 2024). The RBC membrane coating ensures long circulation. Once in the tumor, the enzymes deplete glucose and lactate, starving tumor cells of energy while generating cytotoxic H_2_O_2_. Critically, lactate removal alleviates its immunosuppressive effects (Figure 5B). This metabolic remodeling creates a favorable environment for CpG to exert its adjuvant function, synergistically enhancing dendritic cell maturation and T cell activation. *In vivo*, combining ZLGCR with anti-PD-1 antibody therapy yielded powerful synergistic antitumor efficacy.

In summary, biomimetic and biohybrid strategies have successfully endowed nanoplatforms with “biological identities,” greatly improving targeted delivery and baseline immune activation. However, effective cancer immunotherapy depends not only on where the drug goes, but also on when it is released and how it dynamically interacts with the evolving tumor microenvironment. To achieve spatiotemporally precise control and maximize therapeutic synergy, researchers are now developing “smart” nanoplatforms that respond to specific internal or external triggers. This advancement marks a new era in CpG delivery, one defined by on-demand, context-responsive drug release.

## 4 Stimuli-responsive smart nanoplatforms

**FIGURE 6:**
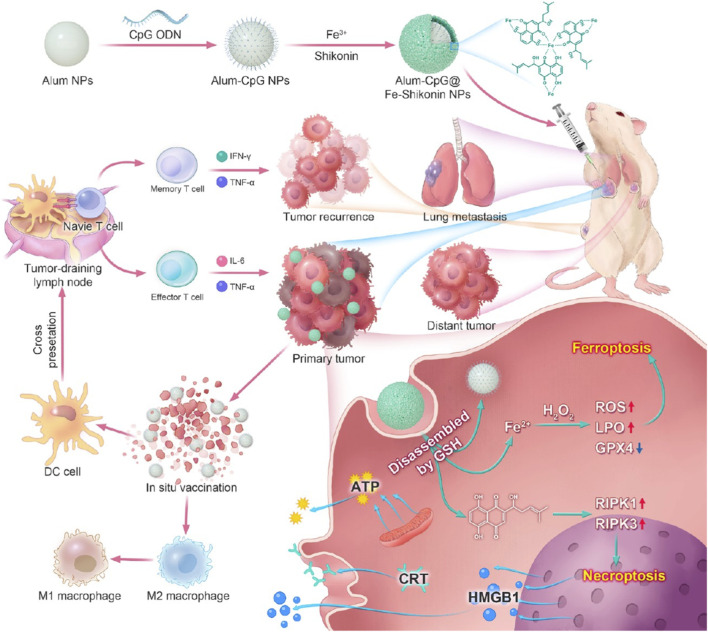
Schematic illustration of the design and immune-mediated antitumor mechanism of the Alum-CpG@Fe-Shikonin nanoplatform. Reproduced with permission from Shi et al. (2023). Copyright (2023), American Chemical Society.

Stimuli-responsive smart nanoplatforms are inspired by the distinct physicochemical differences between tumor and normal tissues, such as acidic pH, elevated reactive oxygen species (ROS) levels, as well as the controllability of external energy fields like light or magnetic fields (Chen et al., 2023; Fang H. et al., 2023; Fang K. et al., 2023; Kuang et al., 2023; Lai et al., 2023; Li X. et al., 2024; Liu et al., 2024; Naghib et al., 2024; Nejabat et al., 2024; Liao et al., 2025). By using these intrinsic or extrinsic cues as triggers, such platforms can achieve spatiotemporally controlled, on-demand release of CpG and other therapeutic agents specifically within tumors. This precise coupling of immune activation with tumor cell killing and ICD greatly enhances both the efficacy and safety of treatment. For instance, Shi et al. developed an *in situ* vaccine platform called Alum-CpG@Fe-Shikonin that integrates responsive release with local immunization (Figure 6) (Shi et al., 2023). In this system, CpG is adsorbed onto aluminum salt nanoparticles, which are then coated with a metal-phenolic network (MPN) made of iron and shikonin. Once internalized by tumor cells, the acidic microenvironment causes the MPN shell to disassemble. The released Fe^2+^ and shikonin work together to induce ferroptosis and necroptosis, two highly immunogenic forms of cell death. The dying tumor cells then release a full set of endogenous tumor antigens, which are efficiently captured by the aluminum core. Antigen-presenting cells subsequently take up both the captured antigens and CpG, initiating a potent, personalized antitumor immune response without the need for *ex vivo* antigen preparation. This “responsive killing to *in situ* antigen capture to co-delivery” design also generated abscopal effects against distant tumors.

**FIGURE 7:**
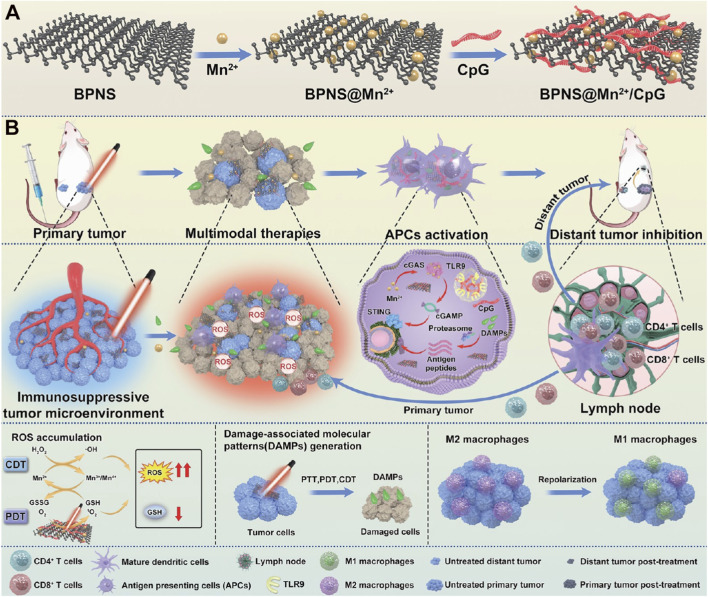
**(A)** Schematic illustration of the synthesis process of the BPNS@ Mn^2+^/CpG nanoplatform. **(B)** Immunotherapeutic mechanism of the BPNS@ Mn^2+^/CpG nanoplatform. Reproduced with permission from Ling et al. (2024). Copyright (2024), American Chemical Society.

While pH-triggered drug release is a widely used strategy, combining it with multi-pathway immune activation and physical therapies can yield even stronger synergy. For example, Jiang et al. engineered a multifunctional platform, BPNS@Mn^2+^/CpG, based on black phosphorus nanosheets (BPNS) (Figure 7A) (Ling et al., 2024). Mn^2+^ ions were coordinated to the BPNS surface to simultaneously stabilize the carrier and load CpG. In the acidic tumor environment, Mn^2+^ and CpG are released. Mn^2+^ activates the cGAS-STING pathway, while CpG stimulates TLR9, two complementary innate immune pathways that together strongly activate antigen-presenting cells (Figure 7B). Meanwhile, under 808 nm laser irradiation, BPNS generates localized heat for photothermal therapy. This thermal effect works with Mn^2+^-mediated chemodynamic therapy to induce robust ICD. The combination of “pH-responsive adjuvant release and light-controlled physical therapy” not only suppressed primary tumors but also established systemic immune memory capable of targeting distant lesions.

Beyond broad ICD induction, stimuli-responsive systems can also be tailored to counteract specific molecular mechanisms of immune escape. Zhao et al. addressed a key challenge in pancreatic cancer: excessive autophagy leads to degradation of MHC class I molecules, allowing tumors to evade T cell recognition (Zhao et al., 2023). To solve this, they designed an ROS-responsive DNA nanoregulator (FNC@NF). This system encapsulates the autophagy inhibitor CQ (chloroquine derivative) within a ferritin core, which is then wrapped in an ROS-cleavable DNA framework containing CpG motifs. In the high-ROS tumor environment, the DNA shell breaks apart, releasing CpG to activate TLR9. At the same time, the ferritin core is actively taken up by tumor cells via transferrin receptor 1 (TfR1). Inside acidic lysosomes, CQ is released and inhibits autophagy, thereby restoring MHC I expression on the tumor cell surface. This “ROS-triggered cascade release” strategy simultaneously boosts immune stimulation (*via* CpG) and reverses a specific immune evasion mechanism (by rescuing MHC I), effectively re-enabling cytotoxic T cells to recognize and kill tumor cells.

**FIGURE 8:**
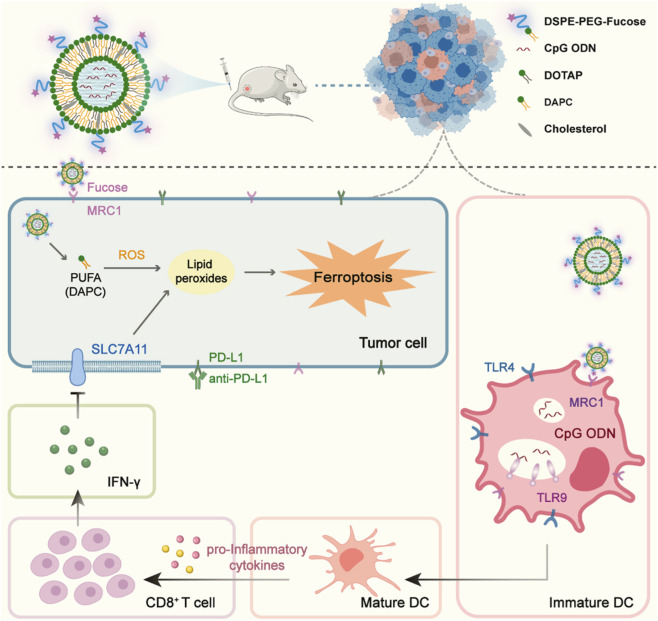
Schematic illustration of the nanostructure and its antitumor mechanisms involving ferroptosis and immunotherapy. Reproduced with permission from Gao et al. (2024). Copyright (2023), Elsevier.

Going a step further, responsive designs can even create self-amplifying therapeutic loops that link two treatment modalities in a positive feedback cycle. Gao et al. developed a unique ROS-responsive liposome to establish such a loop between ferroptosis and immunotherapy (Figure 8) (Gao et al., 2024). The liposome is built from a phospholipid, DAPC, that contains arachidonic acid tails and is loaded with CpG. In the high-ROS tumor environment, DAPC undergoes lipid peroxidation, causing the liposome to rupture and release CpG. The freed CpG then promotes dendritic cell maturation and CD8^+^ T cell activation. Crucially, these activated T cells secrete IFN-γ, which downregulates SLC7A11 in tumor cells, a key component of the cystine/glutamate antiporter. This suppression impairs glutathione synthesis, making tumor cells more vulnerable to ferroptosis. Thus, a self-reinforcing cycle is formed: ROS triggers ferroptosis and CpG release to CpG activates T cells to T cells enhance ferroptosis. When combined with anti-PD-L1 antibody therapy, this system demonstrated remarkable antitumor efficacy.

In summary, stimuli-responsive platforms use sophisticated chemical design to enable intelligent, context-dependent drug release, pushing immunotherapy toward a new level of precision medicine. However, the ultimate strength and quality of the immune response depend not only on how much adjuvant is delivered or when it is released, but also on how it is presented to the immune system. In nature, pathogens potently activate immune receptors through highly ordered, densely packed molecular patterns on their surfaces. Inspired by this principle, the next frontier lies in “structural engineering”: precisely arranging CpG and other immunostimulatory molecules at the nanoscale to maximize their interaction with receptors like TLR9 and thereby amplify immune signal output.

## 5 Structural engineering and spatial arrangement design

Structural engineering goes beyond conventional material chemistry and enters the realm of precision nanofabrication. Its central idea is to precisely control the spatial position, density, and spacing of immune-stimulating molecules, such as CpG, and antigens on the surface of nanocarriers. The spatial co-localization of these two components is not merely a supplementary design but a core factor determining the efficiency of immune activation. Spatial co-localization and nanoscale organization of CpG and antigens (*e.g.*, distance, density, valency) are critical for efficient cross-presentation, DC maturation, and T/B cell activation. Proper spacing enhances TLR9 multivalent binding and sustained immune signaling, directly determining antitumor immunity magnitude and durability. Specifically, appropriate co-localization ensures simultaneous recognition of CpG and antigens by their respective receptors on the same immune cell, enhancing TLR9 activation and antigen presentation to T cells, strengthening antigen-specific T cell function. Moreover, precise density and valency of co-localized CpG and antigens facilitate TLR9 activation, synergistically promoting B cell proliferation, antibody secretion, and memory cell formation. To experimentally validate the aforementioned effects of nanoscale organization and spatial co-localization on immune activation, precise and robust platforms are essential, and DNA origami offers an almost ideal tool for studying how ligand spacing affects biological outcomes. Leveraging this technology, Zeng et al. used square-shaped DNA origami tiles to systematically investigate how the distance between CpG oligonucleotides influences immune activation (Zeng Y. C. et al., 2024). They found that a spacing of 3.5 nm yielded the strongest DC activation, most efficient cross-presentation of antigen, and robust Th1-polarized immunity. This optimal spacing likely enhances multivalent binding to TLR9 receptors. Furthermore, when combined with anti-programmed death-ligand 1 (α-PD-L1) antibody therapy, vaccines built on this precisely spaced DNA origami platform showed significant synergistic antitumor effects in both melanoma and lymphoma models, along with durable T cell memory.

**FIGURE 9:**
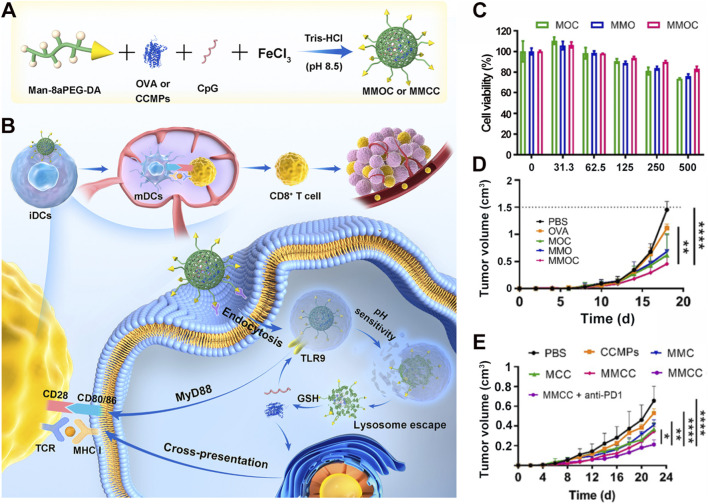
**(A)** Design strategy of MMOC and MMCC. **(B)** Schematic illustration of the immune-mediated antitumor mechanisms of MMOC and MMCC. **(C)** Cell viability after treatment with MOC, MMO, and MMOC at various concentrations. **(D)** Comparison of tumor growth inhibition among different treatment groups in mice. **(E)** Tumor volume changes over time in mice across different experimental groups. Reproduced with permission from Luo et al. (2024). Copyright (2024), Elsevier.

While DNA origami provides exceptional precision, chemically driven self-assembly methods are often more scalable and better suited for multifunctional integration. Metal-phenolic networks (MPNs) represent one such powerful approach. For example, Xue et al. exploited the simple, one-step self-assembly of MPNs to construct mannosylated nanovaccines, termed MMOC or MMCC (Figure 9A) (Luo et al., 2024). These platforms co-loaded either model antigen (OVA) or tumor membrane proteins together with CpG, integrating both components into an ordered network structure. Surface mannose residues enabled efficient targeting of dendritic cells. The MPN framework not only protected the cargo but may also have facilitated optimal co-presentation of antigen and adjuvant. This nanovaccine demonstrated potent antitumor immunotherapeutic efficacy (Figure 9B). The authors assessed cytotoxicity using the CCK-8 assay. Results showed that even at a high concentration of 500 μg/mL, cell viability remained above 70% in the MOC, MMO, and MMOC treatment groups (Figure 9C). *In vivo*, MMOC potently promoted DC maturation and cross-presentation, driving strong antigen-specific CD8^+^ T cell responses in both therapeutic and prophylactic settings (Figure 9D). Furthermore, MMCC combined with α-PD-L1 therapy significantly suppresses tumor growth, attributed to enhancing antigen-specific CD8^+^ T cell responses and DC maturation, and downregulating the proportion of Tregs (Figure 9E).

**FIGURE 10:**
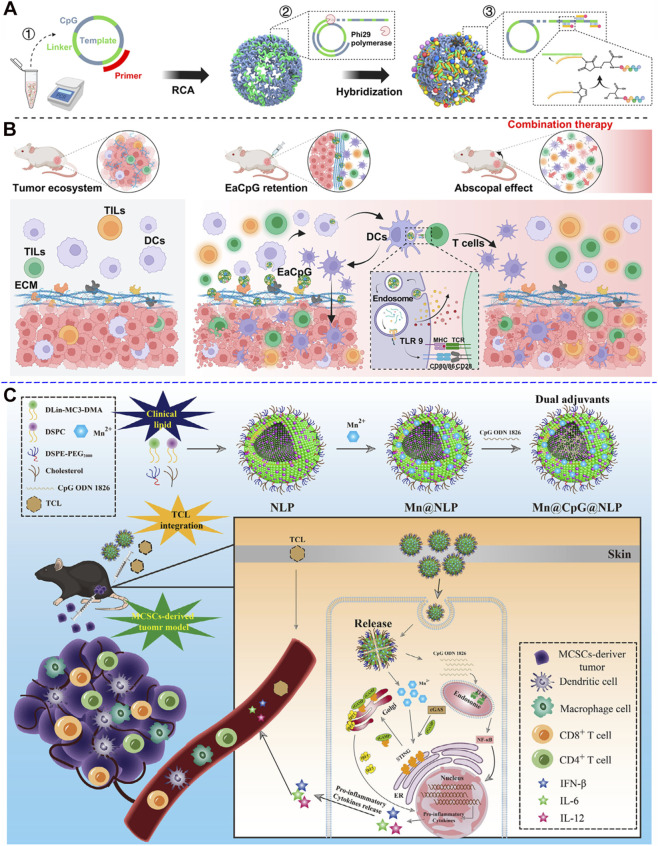
**(A)** Design strategy of the EaCpG nanoplatform. **(B)** Schematic illustration of the immune-mediated antitumor mechanism of the EaCpG nanoplatform. Reproduced with permission from Yang et al. (2023). Copyright (2023), American Chemical Society. **(C)** Schematic illustration of the preparation process and antitumor mechanism of Mn@CpG@NLP. Reproduced with permission from Zhao et al. (2025a). Copyright (2025), Wiley-VCH GmbH.

Structural design can also address pharmacokinetic challenges, such as poor retention of therapeutics at the target site. To overcome the rapid systemic diffusion and short intratumoral residence time of free CpG, Zhang et al. developed a nanoplatform called EaCpG (Yang et al., 2023). They first used rolling circle amplification to generate DNA nanostructures densely packed with multiple CpG repeats. Then, they hybridized ECM-binding peptides onto the surface of these structures (Figure 10A). After peritumoral injection, these peptides anchored the nanoparticles firmly within the tumor extracellular matrix, dramatically prolonging local retention while nearly eliminating systemic leakage (Figure 10B). This sustained, high-concentration local stimulation triggered potent antitumor immunity. Importantly, when combined with radiotherapy or chemotherapy, locally administered EaCpG induced robust systemic immune responses and strong abscopal effects, outperforming free CpG in both efficacy and safety.

Beyond arranging molecules on a carrier’s surface, another key “structural engineering” strategy involves modifying the carrier’s own chemical composition to endow it with intrinsic immunomodulatory functions. Jia et al. demonstrated this by designing a Mn^2+^-doped liposomal platform, Mn@CpG@NLP (Zhao et al., 2025a). Specifically, they incorporated Mn^2+^ ions directly into the lipid bilayer of nanoliposomes (Mn@NLP), which not only imparted a positive surface charge, enhancing cellular uptake, but also turned the carrier itself into an active immune stimulant (Figure 10C). Negatively charged CpG was then adsorbed onto the surface via electrostatic interactions. Critically, the embedded Mn^2+^ acted as a STING pathway agonist, working in concert with CpG to deliver dual adjuvant signals. This synergy significantly enhanced DC maturation and generated effective antitumor immunity in a colon cancer model derived from cancer stem cells.

Through structural engineering, researchers can now “sculpt” immune signals at the nanoscale to fully unlock the activation potential of adjuvants like CpG. However, even the most optimized immune initiation signal may be overwhelmed or exhausted by the highly immunosuppressive microenvironment of established solid tumors. Therefore, true therapeutic breakthroughs often require a coordinated “multi-army” approach. This means CpG nanoplatforms must evolve beyond being excellent “signalers”, they need to become integrated “coordination hubs” that orchestrate multiple therapeutic modalities. By simultaneously engaging multiple targets and pathways, such platforms can systematically reprogram the tumor immune landscape and ultimately drive systemic, curative-level immune responses.

## 6 Combination therapy and systemic immune remodelling

Combination therapy is a cornerstone strategy for tackling complex diseases like cancer (Mengjiao et al., 2021; Wu et al., 2022; Qin et al., 2025). In cancer immunotherapy, integrating CpG-based nanoplatforms with other treatments that act through distinct mechanisms aims to achieve synergistic effects, where “1 + 1 > 2.” This synergy goes beyond direct tumor cell killing. It also enables multi-dimensional intervention across the cancer–immunity cycle: enhancing antigen availability and danger signals, blocking inhibitory checkpoints, improving immune cell metabolism, and recruiting more effector cells. The ultimate goal is to fundamentally shift the immune balance, both locally and systemically, to achieve durable tumor control and long-lasting immune memory.

A prime example of integrated combination therapy comes from Kaneko et al., who developed a multifunctional platform called αPD-L1/CpG@MCL (Kaneko et al., 2025). This system combines magnetic nanoparticles for hyperthermia, covalently conjugated anti-PD-L1 antibodies, and encapsulated CpG oligonucleotides. Under an alternating magnetic field, the magnetic component generates localized heat, killing tumor cells *in situ* and releasing tumor antigens. At the same time, heat triggers the release of both CpG and the antibody. CpG enhances innate immunity by stimulating DC maturation, promoting M1 macrophage polarization, and improving antigen presentation capacity. Complementarily, by blocking PD-1/PD-L1 signaling on tumor cells, αPD-L1 also relieves checkpoint-mediated suppression of DCs and macrophages, further facilitating CTL-mediated cancer cell killing. Together, this triple-action approach led to complete tumor regression in the notoriously resistant B16F10 melanoma model and established robust immune memory capable of preventing tumor rechallenge.

**FIGURE 11:**
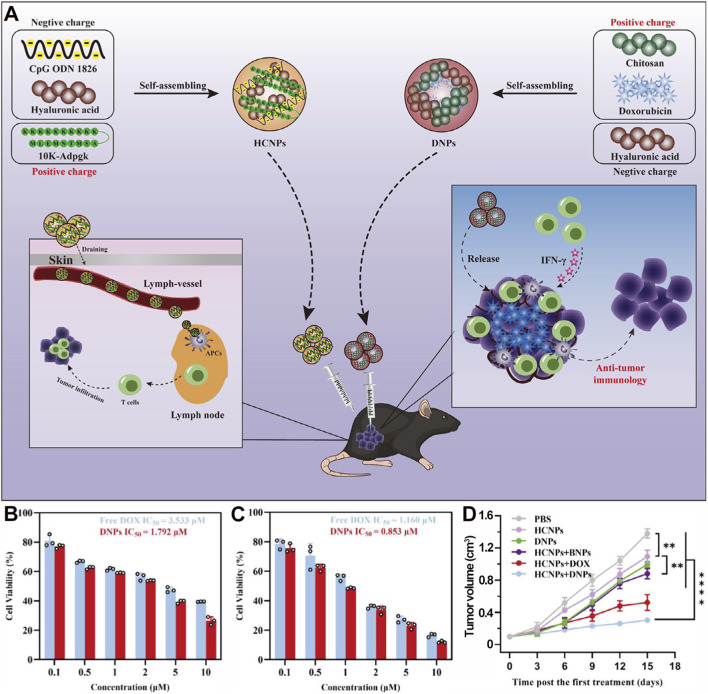
**(A)** Design strategy of the HCNPs and DNPs nanoplatforms. **(B)** Cytotoxicity of DNPs and free DOX after 24 h of treatment. **(C)** Cytotoxicity of DNPs and free DOX after 48 h of treatment. **(D)** Tumor volume changes in mice across different experimental groups. Reproduced with permission from Zhao et al. (2025b). Copyright (2025), Wiley-VCH GmbH.

The synergy between physical therapy and immunomodulation is powerfully illustrated by “thermo-immunotherapy,” while “chemo-immunotherapy” represents a classic paradigm that turns the cytotoxicity of chemotherapy into an opportunity for immune activation. Sheng et al. designed a dual-nanoparticle system to separately deliver immune components and chemotherapeutics (Zhao et al., 2025b). The HCNPs nanoparticle self-assembled to co-deliver a neoantigen peptide (Adpgk) and CpG, specifically targeting dendritic cells for activation (Figure 11A). The DNPs was a pH-responsive doxorubicin (DOX) formulation engineered to target CD44-high tumor cells. DOX-induced tumor cell death triggered ICD, releasing endogenous antigens. These antigens combined with the exogenous antigen and CpG signals from HCNPs to dramatically boost the priming and expansion of antigen-specific T cells. In the MC-38 colorectal cancer model, this combination significantly increased infiltration of CD8^+^ IFN-γ^+^ T cells, reduced M2-like macrophages, and expanded memory T cell pools, resulting in superior antitumor efficacy. Specifically, the authors conducted cytotoxicity assays at 24 h (Figure 11B) and 48 h (Figure 11C). Compared with the DOX-only treatment group, the combination therapy group exhibited significantly lower IC_50_ values, demonstrating enhanced inhibitory effects against MC-38 cells. *In vivo* antitumor efficacy studies further confirmed that co-administration of HCNPs and DNPs achieved the strongest tumor suppression (Figure 11D). Doxorubicin is a widely studied chemotherapeutic agent (Lusha and Mei, 2021; Kang et al., 2022; Liu et al., 2022; Tang et al., 2025; Weng et al., 2025). Its combination with CpG enables potent antitumor efficacy at reduced dosing concentrations, thereby effectively mitigating its inherent systemic toxicity.

This chemo-immunotherapy synergy can be further enhanced by silencing key immunosuppressive genes, thereby sensitizing tumors to chemotherapy and reshaping the microenvironment. Meng et al. addressed the dual challenges of temozolomide (TMZ) resistance and profound immunosuppression in malignant glioma by developing tNano-S&C, a polymer vesicle co-loaded with STAT3 siRNA and CpG (Zhao S. et al., 2025). STAT3 is a master regulator of tumor survival, drug resistance, and immune suppression. Knocking down STAT3 not only sensitized glioma cells to TMZ and promoted ICD but also directly dampened immunosuppressive pathways. Meanwhile, CpG provided a strong activating signal. Both agents were efficiently delivered across the blood–brain barrier *via* apolipoprotein E peptide-functionalized vesicles. Their combined action remodeled the glioma immune landscape, significantly boosting the chemo-immunotherapeutic effect of TMZ. In mouse models, this led to prolonged survival and even complete remission in some animals, offering a new blueprint for chemo-immunotherapy in brain tumors.

**FIGURE 12:**
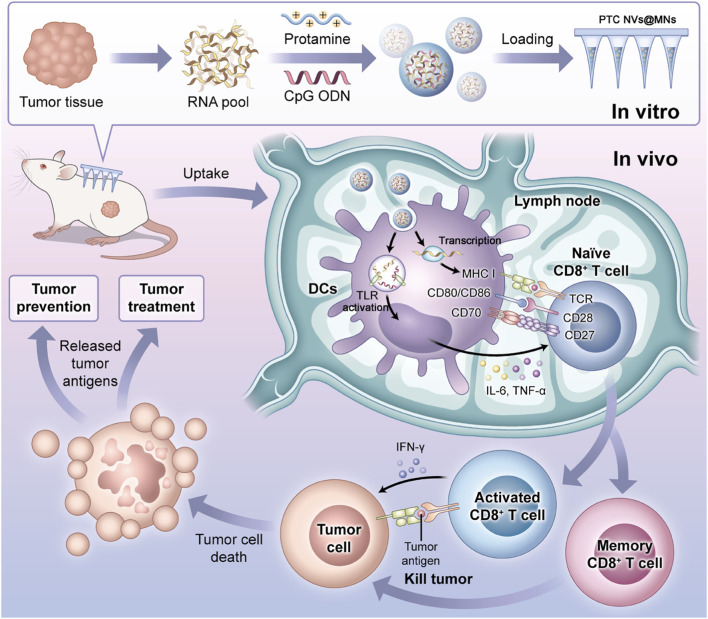
Schematic illustration of the combination of a nanovaccine with nanoneedles and its immune-mediated antitumor mechanism. Reproduced with permission from Wang J. et al. (2025). Copyright (2025), Acta Materialia Inc.

While gene silencing modulates tumor and immune cells from within, combining therapies with better antigen sources provides a broader set of external targets. Total tumor-derived RNA (TdRNA), which contains the full repertoire of patient-specific mutations, is a promising personalized antigen library. Building on this idea, Shuai et al. innovatively used soluble microneedle patches (MNs) to deliver a nanovaccine (PTC NVs) loaded with TdRNA and CpG through the skin (Figure 12) (Wang J. et al., 2025). The microneedles painlessly penetrated the stratum corneum and deposited the vaccine directly into the dermis, an area rich in immune cells. Once inside DCs, TdRNA served two roles: it was translated into a broad spectrum of tumor antigens, and its RNA structure itself acted as a pathogen-associated molecular pattern to stimulate immunity. CpG further amplified this response. This “personalized antigen + classic adjuvant” combination, enabled by stable and efficient microneedle delivery, not only suppressed established tumors but also induced protective immune memory, demonstrating both therapeutic and prophylactic potential.

**FIGURE 13:**
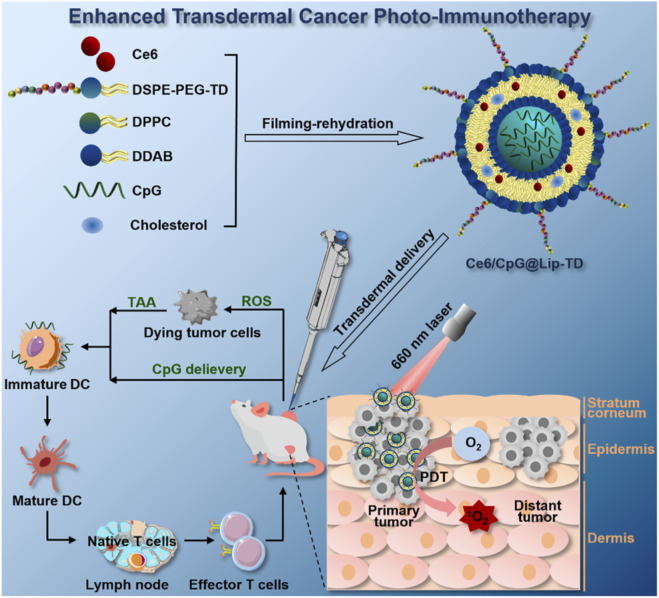
Schematic illustration of the synthesis of the Ce6/CpG@Lip-TD nanoplatform and its combined photodynamic and immunotherapeutic antitumor mechanism. Reproduced with permission from Wang S. et al. (2025).

Microneedles offer an excellent transdermal delivery route (Kang et al., 2022), and for superficial tumors, combining them with localized physical therapies, such as photodynamic therapy, can enable even more precise synergy. In recent years, phototherapy has been widely recognized as a highly effective and precise strategy for cancer treatment (Zhang Q. et al., 2024; Zheng et al., 2026; Gong et al., 2025; Zou et al., 2025; Qi et al., 2024; Ye et al., 2024). Combining phototherapy with immunotherapy not only leverages the strengths of each modality but also induces immunogenic cell death and activates antitumor immune responses, thereby achieving synergistic therapeutic efficacy, truly a powerful combination (Cao et al., 2024; Zhou et al., 2020; Cai et al., 2024; Fan et al., 2024; Yang J. et al., 2024; Zeng B. et al., 2024). Based on this, Li et al. developed Ce6/CpG@Lip-TD, a nanoplatform for topical photo-immunotherapy of skin-accessible tumors (Wang S. et al., 2025). It co-encapsulates the photosensitizer chlorin e6 (Ce6) and CpG within cationic liposomes modified with a transdermal-enhancing peptide (TD) (Figure 13). After topical application, the liposomes effectively penetrate the skin and accumulate in the tumor. Upon laser irradiation, Ce6 generates ROS that kill tumor cells and induce ICD. Simultaneously, the liposome structure breaks down, releasing CpG. The antigens and danger signals from ICD, together with CpG’s immune stimulation, strongly activate both local and systemic antitumor immunity. This leads to suppression of the primary tumor and therapeutic effects against distant metastases. Importantly, this topical, localized combination strategy minimizes systemic side effects and holds strong potential for clinical translation.

## 7 Conclusion

This review systematically summarizes recent advances in intelligent CpG oligonucleotide-based nanoplatforms for targeted cancer immunotherapy and immune remodeling. Core design strategies of CpG nanoplatforms are highlighted, including biomimetic and biohybrid delivery, stimuli-responsive release, structural engineering, and combinatorial therapy. Collectively, these intelligent systems transform CpG from a conventional immunoadjuvant into multifunctional immune regulators that enable targeted delivery, spatiotemporally controlled release, enhanced TLR9 activation, optimized antigen presentation, and systematic remodeling of the immunosuppressive tumor microenvironment. Compared with clinically validated platforms (*e.g.*, mRNA/LNP, protein vaccines), CpG-based nanoplatforms exhibit unique advantages in strong innate immune activation, synergistic compatibility with multiple therapies, and flexibility for *in situ* vaccination, but their translational progression remains relatively limited. This paradigm shift from passive delivery to autonomous immune modulation underscores the potential of CpG nanoplatforms in activating systemic antitumor immunity and establishing long-term immune memory.

Despite substantial progress, current CpG nanoplatforms still face critical drawbacks and translational bottlenecks. First, scalability and cost represent critical translational barriers: most advanced formulations rely on expensive synthetic materials, complex multi-step fabrication, harsh preparation conditions, and low batch yields, which significantly increase production costs, hinder Good Manufacturing Practice-compliant large-scale manufacturing, and severely limit clinical translation. Second, clinical feasibility is constrained by uncertain biosafety profiles, including long-term biocompatibility, *in vivo* degradation, and potential off-target immune stimulation. Third, therapeutic efficacy is highly variable due to tumor heterogeneity, and most platforms are validated only in syngeneic mouse models rather than human tumors. Fourth, batch-to-batch reproducibility is poor, and precise control over CpG intracellular trafficking, release kinetics, and immune cell targeting remains challenging. In addition, compared with mature clinically validated platforms, CpG nanoplatforms lack standardized formulation, clear clinical safety data, and well-defined regulatory pathways, further delaying clinical translation.

To address these limitations and accelerate clinical translation, future research should focus on concrete, clinically oriented solutions. For scalability and cost reduction, designs should prioritize simple structures, low-cost raw materials, modular assembly, and facile preparation without complicated procedures. For improved clinical feasibility, biosafety and biodegradation must be systematically evaluated, and formulations should be optimized for minimal systemic toxicity and controlled immune activation. Deep understandings of tumor heterogeneity and human immune landscapes should be integrated using single-cell omics and spatial transcriptomics to guide rational design. Furthermore, combination strategies with clinically established therapies including radiotherapy, chemotherapy, and checkpoint blockade should be optimized with coordinated timing and delivery routes. Future platforms may also adopt closed-loop feedback control, *in vivo* self-assembly, and integration with mRNA or neoantigen technologies to enhance precision and potency. With rational design and translational consideration, intelligent CpG nanoplatforms hold great promise to become clinically adaptable tools for personalized and effective cancer immunotherapy.
